# Sampling designs for rare time-dependent exposures: a comparison of the nested exposure case-control design and exposure density sampling

**DOI:** 10.1017/S095026882100090X

**Published:** 2021-04-23

**Authors:** J. Feifel, M. von Cube, K. Ohneberg, K. Ershova, M. Wolkewitz, J. Beyersmann, M. Schumacher

**Affiliations:** 1 Faculty of Mathematics and Economics, Institute of Statistics, Ulm University, Ulm, Germany; 2 Institute of Medical Biometry and Statistics, Faculty of Medicine and Medical Center University of Freiburg, Freiburg, Germany; 3 Max Rubner-Institute, Institute of Child Nutrition, Karlsruhe, Germany; 4 Department of Anesthesiology and Pain Medicine, University of Washington, Seattle, USA

## Abstract

In extensive cohort studies, the ascertainment of covariate information on all individuals can be challenging. In hospital epidemiology, an additional issue is often the time-dependency of the exposure of interest. We revisit and compare two sampling designs constructed for rare time-dependent exposures and possibly common outcomes – the nested exposure case-control design and exposure density sampling. Both designs enable efficient hazard ratio estimation by sampling all exposed individuals but only a small fraction of the unexposed ones. Moreover, they account for time-dependent exposure to avoid immortal time bias. We evaluate and compare their performance using data of patients hospitalised in the neuro-intensive care unit at the Burdenko Neurosurgery Institute in Moscow, Russia. Three different types of hospital-acquired infections with different prevalence are considered. Additionally, inflation factors, a primary performance measure, are discussed. We enhance both designs to allow for a competitive analysis of combined and competing endpoints compared to the full cohort approach while substantially reducing the amount of necessary information. Nonetheless, exposure density sampling outperforms the nested exposure case-control design concerning efficiency and accuracy in most considered settings.

Adequate assessment of the clinical and administrative burden of hospital-acquired infections (HAIs) is of major epidemiological interest [1–4]. In a time-to-event analysis, the influence of these exposures on an outcome of interest, often alongside other risk factors, is usually assessed with a Cox regression model. The resulting hazard ratio (HR) relates the hazard of exposed individuals to that of unexposed individuals at the event times. Conventional epidemiological cohort studies require complete covariate information on all individuals irrespective of the exposure and outcome prevalence. The ascertainment of these risk factors is often expensive and may be a huge endeavour. To decrease the economic burden, conventional sampling designs that require covariate information from all patients with an outcome event, but only from a small proportion of individuals without an outcome event have been introduced. More specialised, sampling designs for time-to-event data require covariate information from all patients with an outcome event, but only from a small proportion of individuals without an outcome event. Popular examples are the nested case-control (NCC) [5, 6] and the case-cohort design [7]. Those designs result in competitive estimates compared to the full cohort analysis while at the same time remarkably reducing the necessary resources allocated to the study.

Rare time-dependent exposures but frequent subsequent outcome events are common when assessing the burden of HAIs. In such a situation, nested-case control or case-cohort designs no longer result in the emphasised reduction of resources [8]. Two-phase sampling designs form more informative samples to assess the association of the exposure by exploiting information on the risk factors of primary interest [9, 10]. They even enable the use of cohort (phase I) data in an estimation based on subsampled (phase II) data [11, 12]. Nonetheless, while conventional two-phase designs reduce the required information based on observed outcome events, they frequently result in too large sample sizes [8]. Recently, Ohneberg *et al*. [13] and Feifel *et al*. [8] proposed two schematically different sampling methods, namely exposure density sampling (EDS) and the nested exposure case-control (NECC) design. Being capable of a robust HR estimation while utilizing only limited resources, both NECC and EDS offer researchers an attractive alternative to the expensive full cohort analysis [14] while avoiding one of the most serious types of bias – the immortal time bias [15].

Moreover, in epidemiological studies, cases and controls are often matched on risk factors that are uncomplicated to ascertain. Both exposure-related sampling schemes permit additional matching. Therefore, they enable an estimation that is adjusted for confounders. The model flexibility is increased by matching as assumptions that usually have to be fulfilled for all variables in a statistical model, are not necessary for the matched variables.

This paper revisits NECC and EDS sampling for rare exposures but common and uncommon outcome events. First, we introduce the theoretical background of the two methods. Then, we analyse patients hospitalised in the neuro-intensive care unit (ICU) at the Burdenko Neurosurgery Institute (NSI) in Moscow, Russia [16]. Here, the association of three rare time-dependent infections with different prevalence on length-of-stay in ICU as the outcome event is investigated.

Another frequently observed type of bias is the competing risk bias [17], which can be adequately addressed by both NECC and EDS. To evaluate their performance in this situation, we extend the data analysis to investigate ICU mortality considering discharge alive as a competing risk.

## Methods

**Fig. 1.**
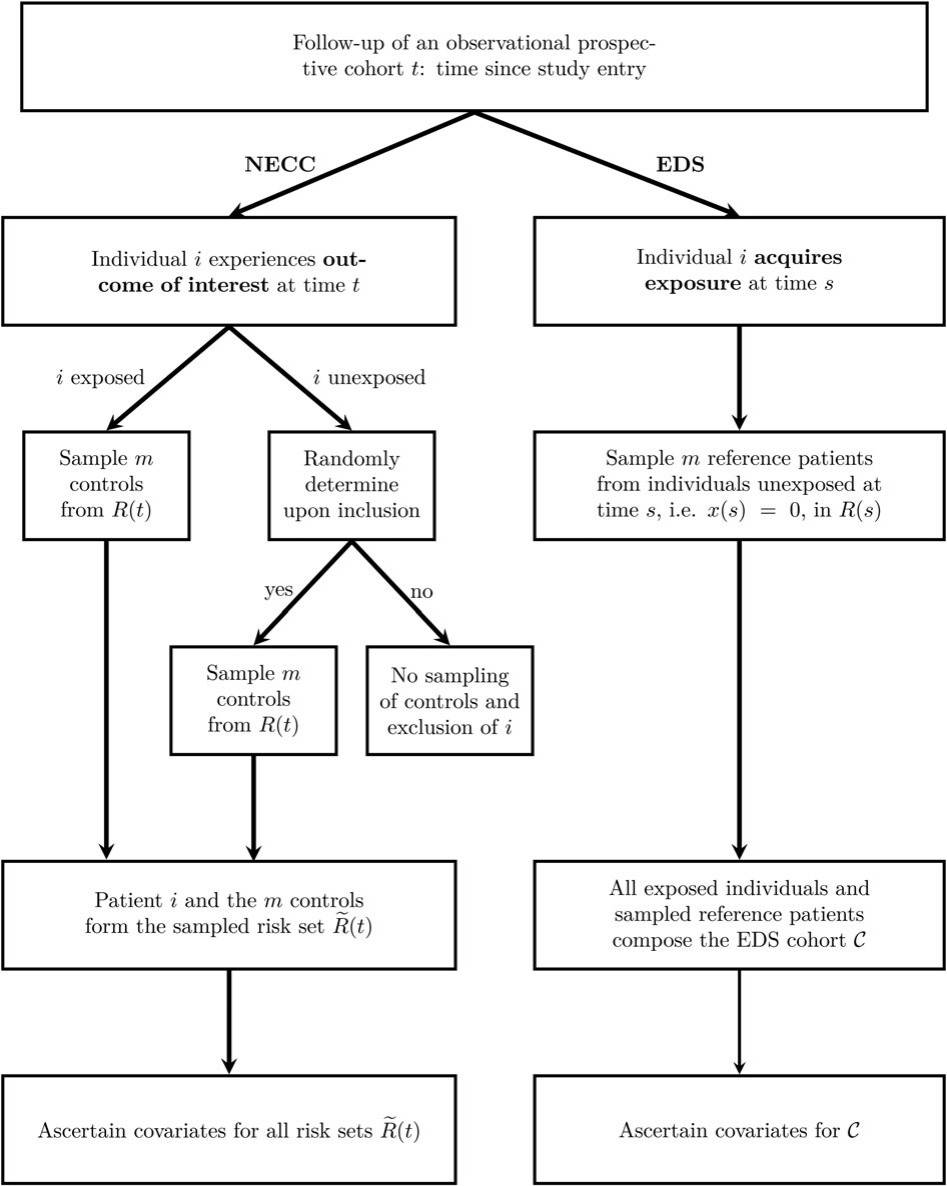
Outline of the two sampling schemes NECC and EDS. The time since study entry is denoted by *t*. The risk set at time *t* for the full cohort is *R*(*t*) and for the NECC cohort is 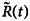. The time-dependent exposure state at time *t* is denoted by *x*(*t*), with *x*(*t*) = 1 indicating that the patient has acquired the exposure by time *t* and *x*(*t*) = 0 indicating otherwise. Finally, 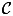 denotes the EDS cohort, which is all exposed individuals and the sampled reference patients. NECC, nested exposure case-control design, EDS, Exposure density sampling.

The two sampling schemes are outlined in Figure 1.

NECC [8] allocates for each exposed individual *i* who experiences the outcome of interest at time *t* a predefined number of controls, denoted by *m*. Additionally, a small fraction (*q*) of unexposed individuals that experience the outcome of interest controls are selected. For both exposed and the randomly chosen unexposed individuals, the controls are sampled at the event times accordingly and information on the risk factors is ascertained. Eligible controls are all individuals that have not experienced the outcome of interest before the time *t* and have not been censored. They are referred to as risk set *R*(*t*). For example, when hospital discharge is the outcome, the *R*(*t*) comprises all individuals with a length of stay at least as long as that of the individual discharged at time *t*. NECC corresponds to the conventional NCC design if *q* is chosen to be one [5, 6].

EDS [13] selects for each exposed individual at time *s*, the time of exposure, *m* unexposed individuals from *R*(*s*) as reference patients. Although showing similarities to controls in nested (exposure) case-control designs, reference patients are allowed to change their exposure status if the exposure is acquired at a later time point than the sampling time. All exposed individuals and all sampled reference patients constitute one cohort, denoted by 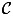. Herein, all individuals are observed until the outcome or a censoring event. The difference compared to a full cohort is, that patients enter with left-truncated entry times. Left-truncated cohorts or delayed entry studies allow for increasing and decreasing risk sets with progressing time. For EDS, the delayed entry takes place upon sampling.

**Fig. 2.**
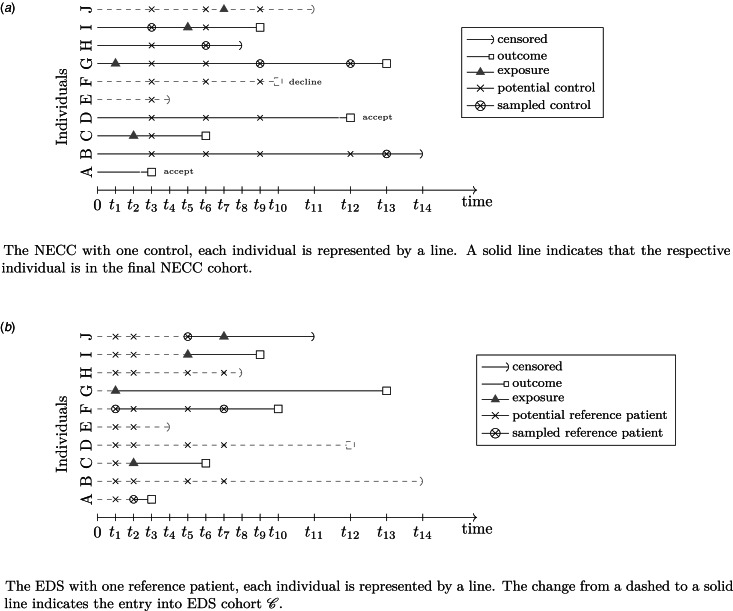
Illustration of the NECC design and the EDS using a fictional cohort of 10 individuals (A–J). NECC, nested exposure case-control; EDS, exposure density sampling.

Figure 2 illustrates the fundamental concepts of the NECC and EDS design on a fictional ICU cohort with individuals A–J. Here, we provide only a brief description of the sampling. A more detailed explanation also including the estimation concept is in the appendix provided in the Supplementary material.

For the NECC design, patient A is included as an unexposed individual with outcome event, and we randomly allocate one control from B, C,…, J, the persons at risk. I is selected and the sampled risk set 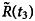 consists of A and I. This concept is similar for patients having an outcome event with prior exposure like C. Here, H and C form the set 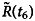. For patients with censored observation (see patient E) or with unobserved outcome event that is not selected, no controls are sampled and they will be excluded.

For EDS design, the time of exposure is essential. The first exposure in the fictional cohort takes place at *t*_1_. Here, the currently unexposed referent F is allocated to patient G, having the exposure event at that moment. The delayed entry time of G and F is *t*_1_. At the time *t*_2_, the exposed individual C and matched patient A enter the cohort. All individuals except for C and G serve as potential matching candidates. The latter is exposed already, therefore, not eligible as a reference patient anymore. Now, the cohort 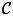 contains the individuals G, F, C, and A. The final EDS cohort 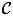 with left-truncated entry times is A, C, F, G, I, and J. The sampling scheme of EDS focuses on exposure times. Thus, individuals with outcome event not yet included in the EDS cohort remain unconsidered.

### HR estimation

A cohort of *n* individuals, where the hazard rate *α*_*i*_(*t*) for each individual *i* follows a Cox proportional hazards [18] model 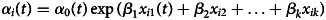, is assumed with the time since admission to the hospital as a time scale. The exposure of primary interest *x*_*i*1_(*t*), is time-dependent and characterises whether the exposure of individual *i* has occurred by time *t*, *x*_*i*1_(*t*) = 1, or not *x*_*i*1_(*t*) = 0. The other covariates are time-independent baseline covariates [19]. The regression coefficients *β*_1_, …, *β*_*k*_ describe the association between the *k* covariates and the baseline hazard. A maximum likelihood estimator for partial likelihood determines the (log-)HR of the Cox regression [20]. At each event time *t*, the covariates of the individual with the outcome event are compared to those of individuals in *R*(*t*). The standard error (s.e.) may be obtained from the inverse of the information matrix.

The estimation of the log HR utilizing NECC and EDS data is also based on a partial likelihood [8]. Both likelihoods are evaluated at the times when the outcome of interest occurred. The sampling and event times coincide for NECC designs. For EDS the sampling time and the time of exposure acquisition are the same.

NECC data can, equivalent to the partial likelihood, be evaluated with a conditional logistic regression as explained in Borgan and Samuelsen [21] and extended in Feifel *et al*. [8]. The NECC analysis is stratified in time with the sampled risk sets 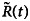 forming the strata. In these sampled risk sets each case is explicitly compared to its allocated controls. The latter aims at approximating all patients still at risk *R*(*t*). This approximation is supported by weights to mimic the multitude of individuals at risk, which usually would have been considered. For each individual *i* the weight is *q* if the individual is currently unexposed and one if not. This weight varies with the exposure status and requires alteration even within individuals.

EDS data are analysed in the same way as a full cohort. The only difference is that the patients enter the analysis with left-truncated entry times. Therefore, both designs respect the time-dependent nature of the exposure.

### Additional matching for sampled cohorts

A stratified version of the Cox model accounts for additional covariates like confounders. Here, the baseline hazard is allowed to differ, whereas the HR of the exposure of interest presumably stays the same for all strata. For an individual *i* the hazard rate in stratum *c* takes the form *α*_*i*_(*t*) = *α*_0*c*_(*t*)exp (*β*_1_*x*_*i*1_(*t*)). For a stratified baseline hazard *α*_0*c*_, restricted sampling of eligible controls or reference individuals is necessary. For NECC, suitable controls are in *R*(*t*) and have the same (or similar) covariates as the individual that experiences the event of interest. For EDS, the eligible referents are all not yet exposed to patients at risk with currently similar covariates as the individual who becomes exposed. This procedure is referred to as additional matching.

The partial likelihood for NECC still applies. The stratification is already adjusted for when considering the sampled risk sets. Thus the estimation of the HR remains the same as before. The analysis of the EDS requires an adjustment on the stratification variable. A stratified Cox regression is comparable to a conventional full cohort approach but accounts for the left-truncated entry times to analyse the EDS cohort.

Both designs allow the matching to be performed only in some of the baseline covariates. The remaining covariates can then be adjusted within the stratified regression model.

### Competing risks

When competing risks are present (for simplicity, we assume two competing events), a cause-specific Cox model is fitted for each competing event. Separate sampling designs are performed as described previously. Now, an individual experiencing event 1 is not at risk for event 2 anymore (and vice versa). In the cause-specific analysis, they are treated similarly to censored individuals. NECC allows for further modifications to enhance the performance at the sampling phase. Different inclusion probabilities *q*_1_ and *q*_2_ can be chosen for each competing event. For example, selecting a larger inclusion probability for rare events compared to common outcomes (see also the results section). The weights for each event type are then calculated using *q*_1_ or *q*_2_, respectively. Different numbers of controls are also possible.

The competing risk scenario mainly affects the estimation stage. NECC considers the sampled risk sets only for the particular event to estimate the HR. Thus, the NECC partial likelihood for one of the competing events considers only the risk sets with the corresponding outcome event. As in the full cohort analysis, the EDS cohort censors all individuals that experience a competing event when focusing on the HR for the event of interest. This analysis is performed for each competing event. An evaluation of the partial likelihood is done at every event time of the particular event type. Nevertheless, as sampling is exposure rather than outcome-based, all unexposed individuals at risk are eligible as reference patients. This implies primarily that the cause-specific Cox regressions for all event types can be performed on the same EDS subsample.

## Results

We analyse patients from the neuro-ICU at the Burdenko NSI in Moscow, Russia. The prospective single-centre cohort study was conducted between 2010 and 2018 and includes 2249 patients who stayed in the ICU for more than 48 h, thus at risk of acquiring an HAI during the ICU stay [16]. In other words, patients enter the study conditional on being in the ICU for at least 48 h. During the follow-up time of 60 days, 685 (30%) patients captured a hospital-acquired urinary tract infection, 211 (9%) a central nervous system infection and 92 (4%) a wound infection. At 60 days, patients are administratively censored (*n* = 122). Among the 2127 patients with an observed event, 304 patients died in the ICU and 1823 were discharged alive. We use NECC and EDS to study the association between different HAIs and the length of ICU stay, the combined endpoint discharge alive and hospital death. The time of infection is considered both in the sampling design and in the Cox regression to avoid immortal time bias. To adjust for confounding by severity of illness, we control for the Charlson comorbidity score by matching or respectively stratification for the full cohort. The two designs are compared to the full cohort and each other using one, two and four controls/reference patients. To show the differences in efficiency and performance, we also present the results of the conventional NCC. For NECC, we use a sampling probability of 10% (i.e. *q* = 0.1) for unexposed individuals who experience the outcome of interest.

To obtain a complete picture of the performance of the two designs and the full cohort approach, we draw 1000 bootstrap samples (with replacement) from the original full cohort data [22]. For each bootstrap sample, we estimate the HR using the full cohort approach, the NECC, the EDS and the NCC with the number of controls/reference patients mentioned above. Bootstrap analysis has several advantages. First, each design's empirical s.e.s can be compared to the mean model-based s.e.s. Second, the results provide not only information on the average performance and efficiency of the sampling designs compared to the full cohort approach, but they also allow for an understanding of how well the various methods (including the full cohort) estimate the true HR of the population [8, 13]. In contrast, a comparison of the designs solely based on the original samples would only allow for a comparison of the NECC's and EDS's estimates and s.e.s with those of the full cohort as reference (gold standard).

We present the mean of the log-HR, the HR and the s.e.s. of the 1000 bootstrap samples. Moreover, we show the mean number of individuals used for the analysis and the mean number of observed events for these individuals. The results for the length of stay are in Table 1. As no differences are observable between the mean model-based s.e.s and sampling s.e.s, we only present the sampling s.e.s.

**Table 1.** Average results based on 1000 bootstrap samples of the original data for the composite endpoint length of ICU stay

HAI (prevalence)	Design	No. sample^a^	No. event^b^	log (HR)^c^	HR	total s.e.^d^
UTI (*P* = 30%)	Referent					
	Full cohort	2249	2127	0.09	1.09	0.06
	Sampling					
	1:1 NCC	2240	2125	0.10	1.11	0.08
	1:1 EDS	985	864	0.10	1.11	0.08
	1:1 NECC	1004	732	0.11	1.12	0.20
	1:2 EDS	1173	1049	0.10	1.11	0.07
	1:2 NECC	1151	730	0.11	1.12	0.17
	1:4 EDS	1408	1284	0.10	1.11	0.06
	1:4 NECC	1328	726	0.10	1.11	0.14
CNSI (*P* = 9%)	Referent					
	Full cohort	2249	2127	−0.32	0.73	0.08
	Sampling					
	1:1 NCC	2240	2125	−0.31	0.73	0.11
	1:1 EDS	393	345	−0.30	0.74	0.11
	1:1 NECC	625	375	−0.28	0.76	0.26
	1:2 EDS	549	488	−0.31	0.73	0.10
	1:2 NECC	804	374	−0.30	0.74	0.19
	1:4 EDS	803	722	−0.31	0.73	0.09
	1:4 NECC	1046	373	−0.29	0.75	0.16
WI (*P* = 4%)	Referent					
	Full cohort	2249	2127	−0.35	0.70	0.11
	Sampling					
	1:1 NCC	2240	2125	−0.34	0.71	0.16
	1:1 EDS	176	147	−0.34	0.71	0.19
	1:1 NECC	492	279	−0.29	0.75	0.36
	1:2 EDS	253	215	−0.34	0.71	0.15
	1:2 NECC	657	278	−0.32	0.73	0.27
	1:4 EDS	390	335	−0.34	0.71	0.13
	1:4 NECC	899	277	−0.32	0.73	0.22

EDS and NECC designs with one, two and four controls/reference patients, NCC design and Cox regressions on the full cohort, have been performed. ICU, intensive care unit; NCC, Nested case-control; EDS, Exposure density sampling; NECC, Nested exposure case-control; CNSI, Central nervous system infection; UTI, Urinary tract infection; WI, Wound infection.

a Number of distinct individuals included.

b Number of events included.

c Adjusted log-hazard ratio of infection.

d Total empirical standard error of log-hazard ratio.

Overall, the differences between the mean HR of the sampling designs and the full cohort are marginal. For the EDS, the largest difference is observed for urinary tract infections (full cohort: 1.09, EDS: 1.11). The largest s.e. of EDS occurs for wound infections with one control/reference patient (s.e. of EDS: 0.19, s.e. of full cohort: 0.11). NECC performs a bit less accurate. The most substantial different HR compared to the full cohort is found for wound infections with one allocated individual (mean HR of NECC: 0.75, mean HR full cohort approach: 0.70). This setting is also the one with the largest s.e., being 0.36 for the NECC. The sample size of the NECC and EDS depends on the prevalence of exposure and the predefined number of sampled controls/reference patients. The higher the prevalence, the more controls/reference patients must be sampled and matched to the exposed patients. As to be expected, the s.e.s decrease with an increasing number of controls/reference patients.

Comparing NECC and EDS, we observe that EDS outperforms NECC in all settings. For exposures with a high prevalence (urinary tract infection), the sample size is comparable. Nonetheless, the s.e.s of EDS are much smaller. For exposures with a low prevalence, estimation with EDS is based on fewer observations. Yet, the s.e.s of EDS remain below those of NECC. The efficient use of controls/reference patients explains this. After sampling with EDS, all reference patients and cases enter the analysis and contribute to the risk set until their event time. In contrast, at each event time, the NECC risk sets are based only on the case, and the controls matched for this specific case. As the outcome is observed for practically all patients, the NCC includes even for the 1:1 sampling almost all patients. Thus, compared to the NCC, the NECC is a more suitable extension of this conventional two-sampling design. The results for NCC designs with more controls are not shown, as they do not provide any additional information.

Regarding the clinical interpretation of the results, we find that both central nervous system and wound infections increase the length of ICU stay. Here, the HR is smaller than one (full cohort 95% confidence interval central nervous system infection: 0.62, 0.85; wound infection: 0.56, 0.88), implying a reduction of the discharge hazard compared to uninfected patients. The sampling designs lead dependent on the number of controls or reference patients *m* to comparable results. Surprisingly, the acquisition of urinary tract infections shows a tendency to be negatively associated with the length of stay (full cohort 95% confidence interval: 0.97, 1.23). The following analysis provides more details on whether this potential decrease is attributable to death rather than earlier discharge alive.

The dataset provides the opportunity to perform a competing risks analysis and to investigate the performance of the two designs for ICU mortality, which is an outcome with a rather low prevalence (≈14%). Thus, we employ two cause-specific Cox regression models, one for discharge alive and one for death in the ICU. To account for the small number of observed deaths, we use a NECC with *q*_1_ of 20% for the cause-specific analysis of in-ICU mortality. For discharge alive in the cause-specific analysis we apply *q*_2_ = 0.1. The results of the competing risks analysis are in Table 2 for the event type death. As most patients are discharged alive (≈81%), the performance of the sampling designs for this event type are similar to the performance observed for length of stay (see Table S1 in the appendix provided in the Supplementary material). Regarding ICU mortality, both sampling designs provide valid approximations of the results for the full cohort approach despite the additional reduced number of observed outcome events. Nonetheless, the results are less accurate than for frequent outcomes. The most substantial difference between the mean HR of EDS and the full cohort occurs for wound infections with one reference patient (EDS: 1.13, full cohort: 1.06). The s.e. is accordingly the highest in this setting (s.e. of EDS: 0.44, s.e. of full cohort: 0.28). However, also the sample size is significantly decreased (EDS: 176, full cohort: 2249).

**Table 2.** Average results based on 1000 bootstrap samples of the original data for the endpoint ICU mortality

HAI (prevalence)	Design	No. sample^a^	No. event^b^	log (HR)^c^	HR	Total s.e.^d^
UTI (*P* = 30%)	Referent					
	Full cohort	2249	304	0.00	1.00	0.16
	Sampling					
	1:1 NCC	529	303	0.01	1.01	0.21
	1:1 EDS	985	137	0.00	1.00	0.20
	1:1 NECC	242	132	0.07	1.07	0.39
	1:2 EDS	1173	165	−0.01	0.99	0.17
	1:2 NECC	334	132	0.04	1.04	0.32
	1:4 EDS	1409	199	−0.01	0.99	0.16
	1:4 NECC	479	130	0.06	1.06	0.29
CNSI (*P* = 9%)	Referent					
	Full cohort	2249	304	0.46	1.58	0.17
	Sampling					
	1:1 NCC	529	303	0.46	1.58	0.26
	1:1 EDS	392	75	0.47	1.60	0.27
	1:1 NECC	194	103	0.57	1.77	0.51
	1:2 EDS	548	94	0.46	1.58	0.22
	1:2 NECC	273	102	0.51	1.67	0.40
	1:4 EDS	802	125	0.46	1.58	0.20
	1:4 NECC	410	101	0.50	1.65	0.31
WI (*P* = 4%)	Referent					
	Full cohort	2249	304	0.06	1.06	0.28
	Sampling					
	1:1 NCC	529	303	0.08	1.08	0.39
	1:1 EDS	176	29	0.12	1.13	0.44
	1:1 NECC	144	75	0.12	1.13	0.61
	1:2 EDS	253	40	0.09	1.09	0.38
	1:2 NECC	206	75	0.18	1.20	0.55
	1:4 EDS	389	57	0.09	1.09	0.32
	1:4 NECC	319	75	0.16	1.17	0.47

EDS and NECC designs with one, two and four controls/reference patients, NCC design and Cox regressions on the full cohort, have been performed. ICU, intensive care unit; NCC, Nested case-control; EDS, Exposure density sampling; NECC, Nested exposure case-control; CNSI, Central nervous system infection; UTI, Urinary tract infection; WI, Wound infection.

a Number of distinct individuals included.

b Number of events included.

c Adjusted log HR of infection.

d Total empirical standard error of log-HR.

Considering the NECC, the most substantial difference compared to the full cohort approach is found for central nervous system infections (mean HR NECC: 1.77, mean HR full cohort: 1.58). The highest s.e. of the NECC again occurs for wound infections with one control (s.e. of NECC: 0.61, s.e. of full cohort approach: 0.28). The loss of power is paid off by an apparent increase in cost-effectiveness. 1:1 NECC uses, on average, only 144 patients for the estimation of the HR.

Compared to EDS, we find that NECC has larger s.e.s in all settings, implying that EDS provides on average more precise estimates of the HR. However, despite a larger inclusion probability chosen for NECC (*q* = 0.2 rather than 0.1), EDS leads to larger sample size for those exposures with a high prevalence (urinary tract infections and central nervous system infections). Given the amount of ascertained covariate information, the NECC may be an attractive alternative here. For urinary tract infections a 1:4 NECC has 45% higher s.e. while using one 49% of the individuals sampled for the 1:1 EDS. Thus, there may be a beneficial trade-off between cost-effectiveness and loss of power when choosing the NECC over the EDS in settings with a rare outcome and a more frequently observed exposure.

The clinical interpretation of the HRs (Table S1 in the Supplementary material) indicates that both central nervous system and wound infections lead to a decreased discharge hazard (full cohort 95% confidence interval central nervous system infection: 0.50, 0.71; wound infection: 0.49, 0.82). The death hazard for central nervous system infections is increased (full cohort 95% confidence interval central nervous system infection: 1.13, 2.21), and thus, the risk of death in the ICU. The association is direct via an increased death hazard and indirect via a decreased discharge hazard. The decreased discharge hazard leads to an extended length of stay, implying that infected patients are longer at risk of dying in the ICU. In contrast, the tendency for a reduced length of stay we previously found for patients acquiring urinary tract infections (or increased for wound infections) cannot finally be explained by a higher (lower) mortality rate. We cannot detect a significant association with the death hazard. In contrast, the discharge-alive hazard is significantly increased, resulting in a decreased length of stay due to faster discharge. However, as we only adjusted for the Charlson score and not, for e.g., other potential infections, we refrain from drawing any clinical conclusion and suspect that additional confounding is still unaccounted.

### Inflation factors

The inflation factor (IF) of an exposure-related cohort sampling method is the ratio of the s.e.s of the sampling design and the full cohort. It quantifies the information obtained in the sampled data compared to the full cohort. An IF of 2 suggests that two-sampled cohorts are of the same statistical power as one full cohort. Especially prior to the sampling, the IF provides a rough assessment of the loss of statistical power. The squared reciprocal IF is referred to as relative efficiency [21, 23]. In studies considering a negligible association between a single exposure and the outcome event, the formula-based IF of NECC is 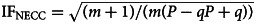. Here, *q* is the NECC's inclusion probability for unexposed individuals who experience the outcome of interest. The number of controls is *m*. The prevalence of the exposure is *P*. As the exposure is time-dependent, *P* refers to the proportion of exposed patients at the end of follow-up. The IF_NECC_ with *q* = 1 equals the IF of the NCC 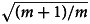. This factor holds independent of the censoring and exposure distribution [24].

Ohneberg *et al*. [13] derived for the EDS 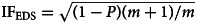. Here, *m* is the number of reference patients per exposed individual. Note the formula-based IF yields a rough approximation, since the time-dependence of the exposure is not taken into account. Additional covariates in the model or large HRs of the time-dependent exposure further alter the proposed designs' efficiency. The precision of the IFs is also affected.

Table 3 shows the formula-based and empirical IFs for the combined endpoint. Dividing the total s.e. of the sampling design by the total s.e. of the full cohort derives the latter. The loss in statistical power for EDS is smaller than for NCC and NECC. Especially, for high prevalence and four controls/reference patients, EDS performs comparably to the full cohort with only 60% of the sample size (1409 *vs.* 2249). Here, the full cohort s.e. for UTI is 0.0613 (refer to Table 1). The s.e. for the EDS with four reference patients is 0.0638 resulting in an IF of 1.0408. The NECC's IF indicates an increase in efficiency compared to the full cohort for decreasing prevalence: the IF is 2.17 for urinary tract infections and 1.92 for wound infections when sampling four controls. Nonetheless, EDS outperforms both the NECC and the NCC, having a smaller IF for the same number of controls/reference patients. For lower prevalence, the IF of 1:1 EDS is smaller than the IF of 1:4 NECC.

**Table 3.** Inflation factor (IF) of the standard errors

HAI (prevalence)	Design	IF empirical	IF formula
UTI (*P* = 30%)	Referent		
	Full cohort	1.00	1.00
	Sampling		
	1:1 NCC^a^	1.40	1.41
	1:1 EDS^b^	1.30	1.18
	1:1 NECC^c^	3.23	2.31
	1:2 EDS	1.12	1.02
	1:2 NECC	2.66	2.00
	1:4 EDS	1.04	0.93
	1:4 NECC	2.17	1.83
CNSI (*P* = 9%)	Referent		
	Full cohort	1.00	1.00
	Sampling		
	1:1 NCC	1.35	1.41
	1:1 EDS	1.47	1.35
	1:1 NECC	3.13	3.29
	1:2 EDS	1.24	1.17
	1:2 NECC	2.43	2.85
	1:4 EDS	1.11	1.06
	1:4 NECC	1.91	2.60
WI (*P* = 4%)	Referent		
	Full cohort	1.00	1.00
	Sampling		
	1:1 NCC	1.36	1.41
	1:1 EDS	1.61	1.38
	1:1 NECC	2.18	3.82
	1:2 EDS	1.34	1.20
	1:2 NECC	2.15	3.31
	1:4 EDS	1.17	1.09
	1:4 NECC	1.92	3.02

The empirical IF is defined as total s.e.(sampling design)/total s.e.(full cohort). For the NECC, we present the results for *q* = 0.1. IF, inflation factor; NCC, Nested case-control; EDS, Exposure density sampling; NECC, Nested exposure case-control; CNSI, Central nervous system infection; UTI, Urinary tract infection; WI, Wound infection.

a NCC design.

b EDS.

c NECC design.

Comparing formula-based to the empirical IF, it becomes apparent that the formulas are merely an approximation of the expected IFs. These estimates are closer to the empirical IFs, if the HR is close to one (see urinary tract infection). The strongest deviation occurs for wound infections. Interestingly, while EDS's formula seems to underestimate the empirical IF, the IF-formula of NECC is more conservative by overestimating the empirical IFs. For EDS the IF may become even smaller than one (urinary tract infection, four reference patients).

## Discussion

The NECC and EDS are parsimonious competitors to a full cohort analysis when analysing rare time-dependent exposures and not necessarily frequent outcomes. They complement the prominent class of sampling design tailored for studying the association between exposures, that may or may not be rare, and rare outcomes.

For data on HAIs, the NECC and EDS reveal a significant decrease in the required resources while providing accurate HR estimates. Case-control designs like the NCC or the NECC stratify for the risk sets and appear to be a natural choice. Nonetheless, in the considered settings with a common outcome, the EDS outperformed the NECC irrespective of the prevalence of exposure. EDS had smaller s.e.s for a lower number of reference patients.

In the analysis of the competing risks, the benefit of EDS over NECC was not as clear. Especially, in settings with a rather rare outcome (ICU mortality) and a rare exposure prevalence NECC uses substantially fewer controls. Thus, even though the EDS results in smaller s.e.s, the NECC may be the preferred sampling scheme in these settings as it provides accurate estimates at meager costs.

In competing risks analysis, the EDS has the advantage that it utilises the same reference patients to study all competing event types. Eventhough this is also possible using the NECC, we show that an adaption of the inclusion probability *q* for the outcome prevalence leads to better results for rare outcomes.

We present IFs, that roughly quantify the loss of statistical power of NECC or, respectively, EDS compared to the full cohort. The IF for the EDS has been already proposed by Ohneberg *et al*. [13], for the NECC, this manuscript gives the first expression. The IFs confirm the observed performance difference between EDS and NECC designs. However, we also noted that the IFs of the EDS are somewhat optimistic, while the IFs of NECC are rather conservative compared to the empirical IFs.

Finally, we conclude that both designs are attractive alternatives to the full cohort analysis. They are both flexible designs that consider the most commonly observed biases: immortal time bias and competing risks bias. Similar to Bang *et al*. [25] and Nelson *et al*. [26], where EDS-like sampling was conducted in cooperation with a propensity score, EDS and NECC allow for propensity score-adjusted matching. In future applications, this adjusted sampling enables comparable control/reference groups that differ only for the exposure and can account for additional confounders. Our data example indicates that overall the EDS results in more precise estimates of the HR. However, for rare outcomes and rare exposures NECC outperformed EDS concerning economic burden while maintaining a dependable estimate of the HR.

## Data Availability

Computing code is available as Supplementary Material. The dataset analyzed during the current study is available in the Zenodo repository, https://doi.org/10.5281/zenodo.3995319.

## Supplementary material

For supplementary material accompanying this paper visit http://dx.doi.org/10.1017/S095026882100090X. click here to view supplementary material
