# Supplementary material for: Sampling designs for rare time-dependent exposures: a comparison of the nested exposure case-control design and exposure density sampling
Source: Epidemiol Infect. 2021 Apr 23;149:e122. doi: 10.1017/S095026882100090X (PMC8165129; doi:10.1017/S095026882100090X)
Supplement: Supplementary file 1 [file S095026882100090Xsup001.pdf]

Supplementary Material

SAMPLING DESIGNS FOR RARE TIME-DEPEN-DENT EXPOSURES - A COMPARISON OF THE NESTED EXPOSURE CASE-CONTROL DESIGN AND EXPOSURE DENSITY SAMPLING

J. Feifel[1], M. von Cube[2], K. Ohneberg[2,3], K. Ershova[4], M. Wolkewitz[2], J. Beyersmann[1], and M. Schumacher[2]

Author affiliations: 1) Institute of Statistics, Faculty of Mathematics and Economics, Ulm University, Ulm, Germany

2) Institute of Medical Biometry and Statistics, Faculty of Medicine and Medical Center University of Freiburg, Freiburg, Germany

3) Max Rubner-Institute, Institute of Child Nutrition, Karlsruhe, Germany

4) Department of Anesthesiology and Pain Medicine, University of Washington, Seattle, USA

Correspondence to Jan Feifel, Institute of Statistics, Ulm University, Helmholzstraße 20, 89081 Ulm, Germany, E-mail: jan.feifel@uni-ulm.de, Tel:+49731 5033102

Running head: Sampling designs for time-dependent exposures

Financial support: This work was supported by grant BE-4500/1-2 from the German Research Foundation.

Declaration of Interest: declared.

Data and Code: Computing code is available as Supplementary Material. Readers can contact the authors if they want access to the data.

APPENDIX

Detailed introduction of the two sampling designs

Nested exposure case-control design

Feifel et al. [1] introduced the nested exposure case-control design. Sampling only a proportion of individuals experiencing the outcome of interst for nested case-control designs and time-fixed covariates was also briefly mentioned in [2]. NECC is an outcome-dependent sampling strategy and can be considered an enhanced nested case-control design. The sampling scheme selects m controls for each exposed individual ($x_i(t) = 1$). Additionally, for all unexposed failing individuals, a random experiment is conducted. A small success probability q determines if the individual is included in the sampled cohort and whether controls are selected. The controls are selected from the risk set $R(t)$ by simple random sampling (without replacement). The sampled individual, including the individuals that experienced the outcome (case) and the controls are referred to as sampled risk set $\tilde{R}(t)$. Exposed individuals supposedly contain more information than unexposed individuals on the relative risk for exposure.

Covariate values are only ascertained for the individuals in the sampled risk sets but are not necessary for the other individuals in the cohort. The reduction in the number of distinct individuals or covariate information compared to a traditional nested case-control design is, therefore, mostly generated by the random experiment.

We summarize the design as

- an individual fails at time t.
  - If the individual was exposed, m controls are sampled from $R(t)$.
  - If the individual was not exposed and the random experiment was successful, m controls are sampled from $R(t)$.
  - If the individual was not exposed and the random experiment was unsuccessful, the individual is excluded and no controls are sampled.
- The case and its controls form the sampled risk set $\widetilde{R}(t)$.

- For all risk sets $\widetilde{R}(t)$ the covariate values are ascertained.

Figure S1A depicts fundamental concepts of the NECC design on a constructed ICU cohort with individuals A to J. All individuals are observed from admission until discharge (alive or dead). NECC is an outcome related sampling design, i.e. one or several controls are sampled at each uncensored event time.

If a patient is leaving the ICU without being exposed, first, a random experiment or a coin toss with success probability q is performed. For illustrative purposes, the inclusion probability q is 67%; usually, it would be prospectively chosen much smaller.

The coin toss for the first patient that has an event, A, is successful, i.e. we randomly allocate one control from B, C,..., J, the persons at risk. Here, I is selected and the sampled risk set $\widetilde{R}(t_3)$ consists of A and I. For E no event is observed due to censoring. Thus no sampling takes place. Patient C is the first individual within our cohort, having an outcome event after prior exposure. This history results in a definite selection of controls; H is chosen. Another typical feature is displayed at time t_9. Individuals that have been selected as controls can become cases later in time. At time t_{10}, an unexposed event with an unsuccessful coin toss is observed. No controls will be sampled and the failing individual F will be excluded. For the next observed event (occurring for patient D) the coin toss is successful. Individual G is again selected as a control. As patient G, the next individual experiencing the outcome event, has been exposed before, we sample a control B. The risk set $\widetilde{R}$ contains G and B. In total, six individuals in five risk sets are includes in the sampled cohort, depicted with solid lines. NECC does not distinguish between exposed or unexposed individuals when selecting controls. Each sampling is performed independently at different event times. Thus a patient can serve as control several times, here control G for failure D and I.

Exposure density sampling

The exposure density sampling is based on dynamical matching for a rare time-dependent exposure [3]. The main focus is estimating the association of the exposure with a more common subsequent outcome event. EDS samples m reference patients for each exposed individual at the time of exposure acquisition. The reference patients are chosen using a simple random sampling among the currently unexposed individuals in $R(t)$. Referents are allowed to become exposed after having been sampled to avoid bias [4]. Dynamical treatment of time-dependent covariates properly accounts for

the waiting period until exposure and averts immortal time bias [5]. The sampling scheme of EDS can also be referred to as matching for the time-to-exposure [6]. From their sampling time or time of exposure acquisition until the end of follow up, the individuals are treated as part of a left-truncated cohort and covariate information is ascertained for them. Time-dependent covariates are observed from the sampling time or left-truncated entry time onward, but not retrospectively. We summarize the design as follows:

- An individual gets exposed at time s.
- m reference patients are sampled from all individuals in $R(s)$ that are not exposed at s, i. e. $x(s) = 0$.
- All exposed individuals and sampled reference patients compose the EDS cohort $\mathscr{C}$.
- Covariate values are ascertained for all individuals in $\mathscr{C}$ from their sampling time until the end of follow-up.

Figure S1B exemplary outlines the procedure on the cohort considered in Figure S1A. The first exposure event happens for individual G at time t_1. At this time point, individual F is selected as reference patient from all eligible individuals (all except G since no other exposure has occurred so far). G and F enter the cohort at this sampling time, which also constitutes their entry time or left-truncation time. The dashed line turning solid at the left-truncation time on the individuals highlights the entry to the cohort. At the time t_2, exposed individual C and referent A enter the cohort. All individuals except for C and G serve as potential reference patients. The latter is exposed already, therefore, not eligible as reference patient anymore. Now, the cohort contains the individuals G, F, C, A. Individual J becomes exposed after the entry to the cohort as a reference patient for individual I. Nevertheless, a referent is assembled for patient J from all at risk and still unexposed, i.e. B, D, F, H. Patient F is sampled for a second time, although this patient is already included. The covariate information of this patient has already been collected. The final EDS cohort with left-truncated entry times is A, C, F, G, I, J. The sampling scheme of EDS focuses on exposure times. Thus, failing individuals not yet included in the cohort remain unconsidered. In the hypothetical example cohort, this is only individual D.

Table S1C outlines for each event time the risk set of NECC, EDS and the full cohort approach.

The two sampling designs can be considered approximations of the latter gold-standard procedure. The risk sets are based on the cohort sampled in Figures S1A and S1B. The partial likelihood for each of the three designs compares covariate information of the patient experiencing the outcome of interest with the information on all individuals in the risk set.

At the event time t_3, the NECC likelihood compares patient A to individual I. Here, it is essential to treat I as an unexposed individual to avoid bias. The full cohort is also comparing A to the unexposed patient I but additionally to patients B-J. The NECC-weight of individual A and I as both unexposed is $w_i = q = 0.67$. Patient I, later gets exposed at time t_5 and will be treated as exposed for all later time points t_6 until t_{13} with a weight of 1. At each event time, the NECC only evaluates the likelihood within the sampled risk set, here one case with its controls.

EDS utilizes a left-truncated partial likelihood to evaluate the sampled data. This analysis is similar to the Cox proportional hazards model. All patients that have been sampled and are still at risk will influence the analysis by appearing in the likelihood. At t_9, an exposed failure I is compared to F, G, I, J and, in contrast to NECC, not only to its reference patient J. The characterization 'reference patient' of the sampling step does not translate to the estimation. The sampled cohort is treated dynamically and reference patients sampled initially can acquire the exposure at a later point and, therefore, appear as 'case'. Patient A influences the estimation as a case, although sampled initially as a reference patient for an exposed C. Once again, all individuals within the sampled EDS cohort, that are still at risk serve as the comparison group.

The components of the estimation are outlined in Table S1C. The succinct feature of an in- and decreasing risk set becomes apparent when comparing the risk sets at t_3 until t_9. At t_6 individual I and G enter the cohort, whereas A is not at risk anymore. Patient G with early exposure and late failure is utilized at all times t_3 until t_{12}. The left-truncated entry times allow for a direct approximation of the EDS cohort on the full cohort without adding weights.

Both designs enable an evaluation of the partial likelihood at different event times, depending on the sampled cohort. EDS evaluates the likelihood at t_{10} and not at t_{12}, the NECC at t_{12} and not at t_{10}.

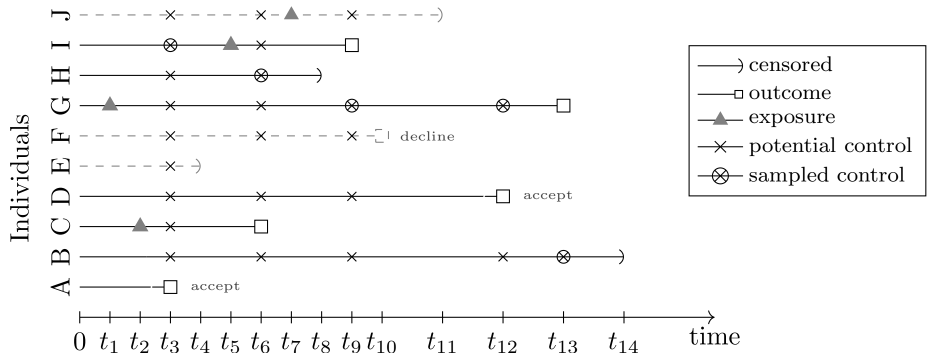

A) The NECC with one control, each individual is represented by a line. A solid line indicates that the respective individuals is in the final cohort.

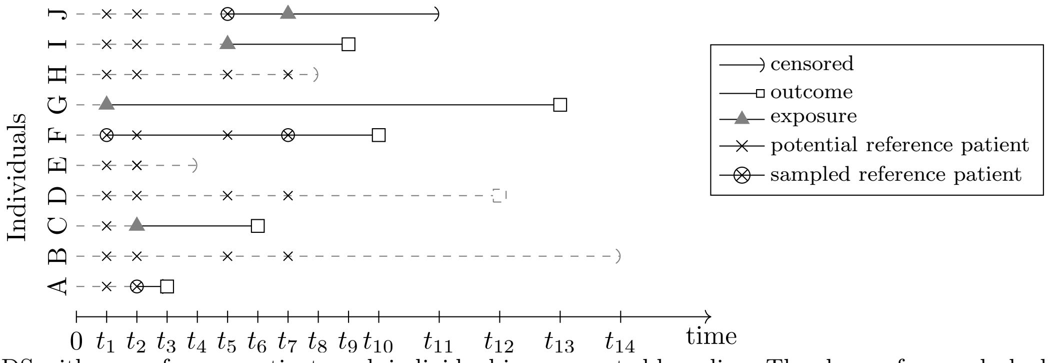

B) The EDS with one reference patient, each individual is represented by a line. The change from a dashed to a solid line indicates the entry of into cohort C.

time	Individual with event	NECC	EDS	Full cohort
t_3	A	A, I	A, C, F, G	A, B, C, D, E, F, G, H, I, J
t_6	C	C, H	C, F, G, I, J	C, B, D, F, G, H, I
t_9	I	I, G	F, G, I, J	I, J, B, D, F, G
t_{10}	F	-	F, G, J	F, B, D, G, J
t_{12}	D	D, G	-	D, G, B
t_{13}	G	G, B	G	G, B

C) Risk sets of the NECC, EDS and the full cohort approach at the observed event times.

Supplementary Figure S1. Illustration of the nested exposure case-control design and the exposure density sampling using a fictional cohort of 10 individuals (A to J).

Additional tables

Supplementary Table S1. Average results based on 1000 Bootstrap samples of the original data for the endpoint discharge alive. EDS and NECC designs with 1,2 and 4 controls/reference patients, nested case-control and Cox regressions on the full cohort, have been performed.

HAI (prevalence)	Design	n.sample[a]	n.event[b]	log(HR)[c]	HR	totalSE [d]
UTI[e] ($p = 30\%$)		Referent				
	Full cohort	2249	1823	0.11	1.12	0.06
		Sampling				
	1:1 NCC	2066	1822	0.12	1.13	0.09
	1:1 EDS	985	728	0.12	1.13	0.08
	1:1 NECC	897	620	0.14	1.15	0.23
	1:2 EDS	1174	886	0.12	1.13	0.07
	1:2 NECC	1054	620	0.11	1.12	0.17
	1:4 EDS	1410	1087	0.11	1.12	0.07
	1:4 NECC	1240	617	0.12	1.13	0.15
CNS[f] ($p = 9\%$)		Referent				
	Full cohort	2249	1823	-0.51	0.60	0.09
		Sampling				
	1:1 NCC	2067	1821	-0.50	0.61	0.13
	1:1 EDS	392	269	-0.49	0.61	0.13
	1:1 NECC	516	297	-0.46	0.63	0.28
	1:2 EDS	549	393	-0.50	0.61	0.11
	1:2 NECC	682	297	-0.48	0.62	0.21
	1:4 EDS	802	596	-0.51	0.60	0.10
	1:4 NECC	922	297	-0.49	0.61	0.17
WI[g] ($p = 4\%$)		Referent				
	Full cohort	2249	1823	-0.45	0.64	0.13
		Sampling				
	1:1 NCC	2067	1822	-0.45	0.64	0.17
	1:1 EDS	176	118	-0.43	0.65	0.21
	1:1 NECC	418	232	-0.35	0.70	0.44
	1:2 EDS	253	175	-0.44	0.64	0.17
	1:2 NECC	569	232	-0.40	0.67	0.32
	1:4 EDS	390	278	-0.44	0.64	0.15
	1:4 NECC	802	232	-0.41	0.66	0.24

[a] number of distinct individuals included;
[b] number of events included [c] adjusted log hazard ratio of infection;
[d] total empirical standard error of log-hazard ratio; [e] urinary tract infection;
[f] central nervous system infection; [g] wound infection

REFERENCES

[1] Feifel J, Gebauer M, Schumacher M, et al. Nested exposure case-control sampling: a sampling scheme to analyze rare time-dependent exposures. *Lifetime Data Analysis*. 2020;26(1):21–44.

[2] Langholz B, Borgan Ø. Counter-matching: A stratified nested case-control sampling method. *Biometrika*. 1995;82(1):69.

[3] Ohneberg K, Beyersmann J, Schumacher M. Exposure density sampling: Dynamic matching with respect to a time-dependent exposure. *Statistics in Medicine*. 2019;38(22):4390–4403.

[4] Savignoni A, Giard C, Tubert-Bitter P, et al. Matching methods to create paired survival data based on an exposure occurring over time: a simulation study with application to breast cancer. *BMC medical research methodology*. 2014;14(1):83.

[5] Karim ME, Gustafson P, Petkau J, et al. Comparison of statistical approaches for dealing with immortal time bias in drug effectiveness studies. *American Journal of Epidemiology*. 2016;184 (4):325–335.

[6] Wolkewitz M, Beyersmann J, Gastmeier P, et al. Efficient risk set sampling when a time-dependent exposure is present. *Methods of Information in Medicine*. 2009;48(5):438–443.
